# Notes on a Case of Blackwater Fever

**Published:** 1904-06

**Authors:** E. Cecil Williams

**Affiliations:** Physician in Charge of Out-patients, Royal Hospital for Sick Children, Bristol


### NOTES ON A CASE OF BLACKWATER FEVER.

BY

E. Cecil Williams, B.A., M.B. Cantab.,

*Physician in Charge of Out-patients, Royal Hospital for Sick Children, Bristol.*

---

The following are notes of a case of Blackwater fever, occurring in this country three weeks after arrival from West Africa:—

The patient had been, for the greater portion of the last nine years, engaged in mining operations in Ashanti, during which time he repeatedly suffered from malarial fever of the daily type. He had been very careless in adopting the usual antimalarial precautions, viz., routine doses of quinine and sleeping under a mosquito net. He did not always take quinine even during the attacks. So severe, however, were the malarial attacks during the last three months of his residence on the coast that he decided, on medical advice, to return to England, where he landed on December 15th of last year, feeling better for the voyage. On January 5th of this year he got wet and went home to bed. Next morning, January 6th, feeling much better, he went downstairs. Soon after breakfast he had a rigor and vomited; sickness continued all day.

When I saw him at 2 p.m. the vomit was dark green, pulse 120, temperature 103°. His skin was yellow, conjunctiva slightly tinged, mucous membrane of lips very anæmic.

There was nothing abnormal to be heard in the chest; there was tenderness over the liver; pressure made him feel sick; no enlargement of the liver or spleen. He complained of pain in both loins. The urine was dark red and thick. Under the microscope the urine showed a fibrinous network with black pigment spots scattered here and there. There were no casts and no red blood corpuscles. Bowels had been opened twice during the morning; peasoup motions. I gave the patient five grains of quinine. He had a restless night. Next day, January 7th, sickness continued; urine still red but lighter colour; temperature 99°, pulse 104; had a better night. January 9th, morning urine just tinged; temperature 99°, pulse 112. At midnight the urine was clear; all the bad symptoms were rapidly subsiding; in three weeks the patient was well.

In this case I had the valuable advice of Mr. Walter Fisher, who has, during a residence of eight years on the Zambesi, treated thirty cases of this disease with only two deaths. Mr. Fisher always uses quinine hypodermically, and is convinced of its value. He injects five grains once daily; by this method the vomiting is not aggravated. He lays equal stress on keeping the bowels thoroughly well open with small daily doses of calomel, and giving a soap and water enema once or twice daily so long as the peasoup motions continue, in order to keep the large bowel empty and so prevent any absorption of poisonous matter.

Mr. A. R. Sieveking, late principal medical officer to the Uganda Railway, tells me he treated during six years fifty cases of Blackwater fever, and is convinced of the value of hypodermics of quinine.

The disease only occurs in those who have been debilitated by malaria; it may come on in this country many months after leaving Equatorial Africa, and may even prove fatal. The disease has apparently become much more prevalent in Equatorial Africa with the march of civilisation. The turning up of virgin soil, during the construction of railways and other public works, seems to let the specific poison free. The exact nature of the poison is unknown. Authorities differ as to the advisability of giving quinine, some even asserting it aggravates the tendency to hæmolysis. Considering, however, that the subjects of Blackwater fever have always suffered from malaria, quinine seems a rational drug to give, and the experience of Mr. Fisher and Mr. Sieveking in different parts of Equatorial Africa certainly supports this contention.

### NOTE ON THE CONDITION OF THE BLOOD CORPUSCLES IN DR. E. C. WILLIAMS' CASE.

By J. M. FORTESCUE-BRICKDALE, M.A., M.D. Oxon.

THE blood films prepared from this case were not taken till after the subsidence of the fever; they were fixed immediately and stained with Jenner's blood stain or eosin and methylene blue. As is not unusual, malarial parasites could not be found, but the corpuscles showed several interesting features. The formation of rouleaux had recommenced, showing that the attack was subsiding, and poikilocytes, though present, were not numerous. The majority of the erythrocytes were deficient in hæmoglobin, the centre showing a more or less circular area of faint blue colour. The exact significance of this appearance is doubtful, but it seems probable that it is due to some destructive process possibly antecedent to the extrusion of blood-platelets. The latter, however, were not observed. The change is apparently distinct from "polychromasia" and allied to the granular degeneration of Grawitz. This appearance was also well seen in the present instance, many corpuscles being dotted with fine granules. Though there is some divergence of opinion among cytologists on the point, it is clear that this phenomenon occurs in many anæmic conditions (especially post-malarial hydræmia), and is possibly produced by karyorhexis, or the breaking down of the nuclei, especially in megaloblasts. It is noteworthy that some corpuscles showing granular degeneration here were of large size. There was a large number of small basophil bodies lying free in irregular-shaped groups. They were spheroidal or elongated in shape, and represented the bodies known as "blood dust" or hæmokonia. They are variously regarded as products of leucocyte degeneration, of the fragmentation of erythrocytes, or of albuminous particles precipitated from the plasma. The leucocyte count showed a remarkable increase in small mononuclears at the expense of polynuclears, the numbers being about equal. The eosinophils were only .75 per cent. and the large mononuclears about normal (5.5 per cent). Badly stained corpuscles and deformed fragmented leucocytes ("shadow cells") were observed. The nuclei in the polynuclears were frequently entirely separate. Generally, then, the cells both red and white showed extensive degenerative changes, but the formation of rouleaux showed that *restitutio ad integrum* had begun.

### Progress of the Medical Sciences.

### MEDICINE.

